# The potential of lunasin extract for the prevention of breast cancer progression by upregulating E-Cadherin and inhibiting ICAM-1

**DOI:** 10.12688/f1000research.55385.1

**Published:** 2021-09-08

**Authors:** Kusmardi Kusmardi, Elvan Wiyarta, Numlil Khaira Rusdi, Andi Muh. Maulana, Ari Estuningtyas, Hadi Sunaryo

**Affiliations:** 1 Department of Anatomic Pathology, Faculty of Medicine, Universitas Indonesia, Salemba Raya Street no.6, Jakarta, 10430, Indonesia; 2 Drug Development Research Cluster, Indonesian Medical Education and Research Institute, Universitas Indonesia, Salemba Raya Street no.6, Jakarta, 10430, Indonesia; 3 Human Cancer Research Cluster, Indonesian Medical Education and Research Institute, Universitas Indonesia, Salemba Raya Street no.6, Jakarta, 10430, Indonesia; 4 Faculty of Medicine, Universitas Indonesia, Salemba Raya Street no.6, Jakarta, 10430, Indonesia; 5 Faculty of Pharmacy and Science, Universitas Muhammadiyah Prof. DR. Hamka, Limau II Street, Jakarta, 12130, Indonesia; 6 Doctoral Program for Biomedical Sciences, Faculty of Medicine, Universitas Indonesia, Salemba Raya Street no.6, Jakarta, 10430, Indonesia; 7 Faculty of Medicine, University of Muhammadiyah Purwakarta, KH. Ahmad Dahlan Street, Central Java, 53182, Indonesia; 8 Department of Pharmacology and Therapeutics, Faculty of Medicine, Universitas Indonesia, Salemba Raya Street no.6, Jakarta, 10430, Indonesia

**Keywords:** Lunasin, ICAM-1, E-Cadherin, Breast Cancer, Cancer Prevention

## Abstract

**Background:** Research in natural substances for their anticancer potential has become increasingly popular. Lunasin, a soybean protein, is known to inhibit cancer progression via various pathways.  The aim of this study was to investigate the effect of Lunasin Extract (LE) on the expression of Intercellular Adhesion Molecule 1 (ICAM-1) and epithelial cadherins (E-Cadherin) in breast cancer. **Methods: **In this true-experimental *in vivo* study, 24 Sprague-Dawley rats that were induced by 7,12-Dimethylbenz[a]anthracene (DMBA), were used. Based on the therapy given, the groups were divided into, normal, positive control (PC), negative control (NC), adjuvant, curative, and preventive. Lunasin was extracted from soybean seeds of the Grobogan variety in Indonesia. Tissue samples were obtained, processed, stained with anti-ICAM-1 and anti-E-Cadherin antibodies, examined under a microscope, and quantified using H-score. The data were analyzed using ANOVA, which was then followed by Duncan's test. **Results:** Statistically significant difference in ICAM-1 expression was observed between the following groups: adjuvant and NC, normal and NC, PC and NC, adjuvant and preventive, normal and preventive, PC and preventive, adjuvant and curative, normal and curative, PC and curative. E-Cadherin expression was significantly different between preventive and NC, adjuvant and NC, PC and NC, normal and NC, adjuvant and curative, PC and curative, normal and curative, normal and preventive. Significant negative correlation was found between ICAM-1 and E-Cadherin [-0.616 (-0.8165; -0.283)] with p = 0.001. **Conclusion:** Preventive dose of LE was able to reduce ICAM-1 expression while increasing E-Cadherin expression.

## Introduction

Breast cancer is the most frequent type of cancer in women across the world. ^1^ In 2012, according to statistics from the Global Burden of Cancer Study, an estimated 1,671,149 new cases of breast cancer and 521,907 breast cancer deaths occurred in women worldwide, resulting in a prevalence of 25.1% of all cancers. ^1^ The 2018 Globocan statistics indicated that there was an 11.6% decline in the prevalence of breast cancer compared to the 2012 Globocan data. However, the prevalence of breast cancer remains the greatest in women. ^2^

Breast cancer, in specific a metastatic subtype, is an intricate and multi-staged malignant tumor. ^3^ A study by Müllar et al. ^4^ has shown that alterations in the intercellular adhesion molecules can play a vital role in the metastasis of breast cancer. Intercellular Adhesion Molecule 1 (ICAM-1) as a surface glycoprotein, functions as an adhesion molecule on tumor cells that could rearrange actin, promote pro-inflammatory cascade, and mediate multiple signaling pathways to regulate cell metastasis. ^5^ Although ICAM-1 expression varies depending on the breast cancer subtype, ^6^ its expression is significantly increased, especially in metastatic breast cancer. ^7^ Different from ICAM-1, epithelial cadherins (E-Cadherin) is a glycoprotein that functions as an adhesion protein between cells to maintain tissue integrity. ^8^ Therefore, low expression of E-Cadherin is associated with the progression of epithelial-mesenchymal transition (EMT) in breast cancer. ^8^ Due to the high prevalence of this disease, developing breast cancer treatments have become a priority. Cancer is now treated with surgery, chemotherapy, radiation, and targeted therapy, ^9^ which have negative side effects. Chemotherapy as one of the most common cancer treatments has many significant side effects such as mucositis, neurotoxicity, chemotherapy-induced peripheral neuropathy, reduced renal function, and bone marrow depression. ^10^ Many investigations are now developing cancer treatments using natural substances that might be capable of reducing these negative side effects. ^11^

Over the last several decades, an increasing number of plant-derived products, including soybean [ *Glycine max* (L.) Merr.], have been explored, as it is cheap, easy to grow, and abundant in nature. ^12^ Soy products are also known to lower the incidence and mortality of breast cancer, prostate cancer, colon cancer, and lung cancer. ^13- 15^ The molecule in soybeans that works as an anti-cancer is considered to be a bioactive peptide called lunasin. This peptide was first discovered from a sample of commercially accessible soybean germplasm, originating in the United States. ^16^ Lunasin is a lipophilic peptide component made up of 44 amino acids that have anti-cancer, anti-oxidant, and anti-inflammatory properties, as well as the ability to control cholesterol biosynthesis in the body. ^17, 18^

The challenges with synthetic lunasin are that the commercial price is quite high due to its costly and time-consuming synthesis process. Additionally, effective techniques for obtaining a significant amount of pure lunasin from plant sources do not exist. ^19^ Although Lunasin Extract (LE) from the Grobogan type has been shown to have anti-inflammatory and anti-cancerous effects in the colon, studies into various anti-cancer pathways of LE are required to better understand its molecular effect on breast cancer. ^12^

As such, this study aims to investigate the anti-cancer effects of LE from the Grobogan variety in Indonesia. We hypothesized that LE administration could suppress breast cancer progression by decreasing ICAM-1 expression while increasing E-Cadherin expression. By examining the effects of LE on these two molecules, this study is expected to investigate the potential of LE for the prevention of breast cancer progression via ICAM-1 and E-Cadherin expression pathway.

## Methods

### Lunasin extraction

Soybean seeds (Glycine max (L.) Merr) of Grobogan variety were obtained at the Research Institute for Various Nuts and Tubers, Malang, East Java. Soybean seeds undergo the process of defatting in several stages. These seeds were washed with water, and ground using a 3 mm grinder (AEG type AMEB 80 FX). The powder was then wrapped in gauze and pressed at 100-150 atm for 30 minutes at 120°F, to create plates.

The plates are turned into powder by using a mortar and pestle, as well as a blender. The powder obtained was sieved using a mesh sieve (Size 40) and placed into a plastic container and stored at 4°C until the maceration process. Extraction was carried out by the maceration process ^11^ with the use of Phosphate Buffer Saline solvent (PBS) at pH 7.4 (Abcam, ab270748). Soybean powder was added with PBS, stirred until homogeneous, and macerated for 60 minutes with occasional stirring. The maceration product was filtered using a Whatman 54 filter paper. The filtrate was collected and stored at 4°C. Finally, the filtrate was then dried using a rotary evaporator to obtain a thick extract.

Standardization was performed for the viscous extract. The parameters included water content, acid insoluble ash content, heavy metal contamination, and microbial contamination. In this study, standardization of phytochemical components was also carried out, which included tests for alkaloids, flavonoids, saponins, steroids, polyphenols, tannins, and triterpenoids.

The levels of lunasin concentration in the thick extract were determined after some of the parameters were standardized. This process was done by dissolving 100 mg of thick extract in 8 mL of distilled water. The solution was sonicated for 30 minutes, and distilled water was added to a final volume of 10 mL (10,000 ppm solution), then centrifuged at 12,000 rpm for 30 minutes. The resulting supernatant was filtered through a 0.22 mm millipore sieve, resulting in a clear and colorless solution. High-performance liquid chromatography was used to determine the solution content. Extracts that provide higher concentrations of dissolved lunasin were used as LE in this study.

### Study design and sample calculation

This study is a true-experimental *in vivo* laboratory study, which included Sprague-Dawley rats induced by 7,12-Dimethylbenz [a] anthracene (DMBA). This research was conducted from December 2020 to June 2021 at the Molecular Biology and Proteomics Core Facilities, Faculty of Medicine Universitas Indonesia Laboratory. The experimental protocols were approved by the Ethics Committee of the Faculty of Medicine, the University of Indonesia with protocol number KET-647/UN2.F1/ETIK/PPM.00.02/2019.

The experimental animals used in this study were female white rats (Sprague-Dawley) obtained from the National Agency for Drug and Food Control (Indonesia), aged 4-6 weeks, with bodyweight (BW) ranging from 60-80 grams. During the experiment and the analysis, no rats were excluded due to illness or any other reasons. Breast cancer induction was done using DMBA (Merck, D3254) at a dose of 20 mg/kg BW. All samples (n = 24) were divided into six groups consisting of four rats each. The groups were divided into normal, negative control (NC), positive control (PC), curative, adjuvant, and preventive. The sample size was determined by Federer’s formula.

Federer’s formula: (T-1) (N-1) > 15

T = Number of groups

N = Number of rats

All samples were randomly allocated in each group using a random number generator. To avoid bias, the grouping data was only accessed by one researcher (E.W.). Other researchers were blinded until the research process was completed.

Prior to the experiment, all samples in each group were acclimatized for 1 week. Then, each sample received a different treatment depending on their respective groups. The Normal group did not receive any treatment. The rats in the NC group only received DMBA induction until the end of the experiment. In the PC group, DMBA-induced rats with a tumor volume of 1-2 cm ^3^ were given Tamoxifen (Merck, 579002) (10 mg/kg BW) for 8 weeks. In the curative group, DMBA-induced rats with a tumor volume of 1-2 cm ^3^ were given LE (500 mg/kg BW) for 8 weeks. The adjuvant group, DMBA-induced rats with a tumor volume of 1-2 cm ^3^, were given a combination of LE (500 mg/kg BW) and Tamoxifen (10 mg/kg BW) for 8 weeks. The rats in the preventive group received LE (500 mg/kg BW) in week 1, followed by DMBA. Then they were given LE (500 mg/kg BW) until the end of the study. Tumor volume was calculated using the formula: (length) × (width2)/2. ^20^ The total experiment lasted for 24 weeks.

After being euthanized, paraffin blocks of breast cancer tissue from each rat were made for immunohistochemistry (IHC) staining. Unreliable paraffin blocks (e.g., broken/damaged paraffin blocks and/or paraffin blocks where the tumor mass was eaten by animals) were excluded.

### Ethics approval

The experimental protocols were approved by the Ethics Committee of the Faculty of Medicine, University of Indonesia with protocol number KET-647/UN2.F1/ETIK/PPM.00.02/2019. The treatment and maintenance of the animals are following the Guide for the Care and Use of Laboratory Animals by the Animal Care and Use Committee, namely by monitoring the temperature of 25°C, 12 hours of light-dark cycle, 55% humidity, as well as standard food and drink. ^20^ Anesthesia and euthanasia procedures are performed according to the American Veterinary Medical Association (AVMA) Guidelines for the Euthanasia of Animals. ^21^ Anesthesia was performed with ketamine (Merck, NMID686C) at 75-100 mg/kg BW and xylazine (Merck, X1126) at a dose of 10 mg/kg BW intraperitoneally. All efforts were made to ameliorate any suffering of animals. All results are reviewed and reported following the reporting guidelines for animal pre-clinical studies, namely the Animal Research: Reporting of in vivo Experiments ( ARRIVE guideline). ^22, 23^

### Tissue handling and IHC staining

Breast cancer tissue in each sample was placed in a formalin buffer solution (Merck, HT501128), and cut into 3-5 mm thickness with a scalpel. Tissue samples were immersed in 96% ethanol (Merck, 443611) for 30 minutes, this process was repeated five times. The tissue was immersed in the xylol (Merck, 108297) solution for 15 minutes. The tissue was planted in solid paraffin with a melting point of 60-70°C for 30 minutes and placed in a 4 ^o^C freezer for 10-15 minutes. The tissue in the paraffin block was cut using a microtome with a thickness of 3 mm and placed on a glass slide that had been coated with poly-L-lysine and heated in an oven at 60°C overnight. The tissue samples were deparaffinized with xylene three times for 3 minutes each and rehydrated with 100%, 95%, and 70% ethanol for 2, 2, and 1minute, respectively. The tissue samples were immersed in 0.01 M citrate buffer (Merck, 21545) pH 6.0 in the microwave for 5 minutes, and dripped with 3% hydrogen peroxide to remove endogenous peroxide for 5 minutes at room temperature. Each tissue sample was incubated with anti-ICAM-1 antibodies (MyBiosource, MBS2543949) and anti-E-Cadherin antibodies (Abcam, ab134047) in PBS for 2 hours at room temperature in a humidity chamber followed by overnight incubation at 4°C. The N-Universal negative control (Merck, 939B) was used as a negative control. The tissue samples were then incubated with the Novolink Polymer DS 250 test (Novolink, RE7140-CE) for 1 hour at room temperature, followed by incubation for 30 minutes with horseradish peroxidase-conjugated streptavidin (Abcam, ab7403). Proteins were visualized using 3,3′-diaminobenzidine (DAB) for 10 minutes at room temperature, which makes the expression of ICAM-1 and E-Cadherin observable based on the intensity of the brown color. Finally, hematoxylin-eosin counterstain, dehydration, and mounting of the tissue were performed.

### Quantification of ICAM and E-Cadherin expression

Each tissue was observed using a light microscope at a total magnification of 400× and documented using a computer with Leica Application Suite (LAS) EZ V3.0.0 software (Leica Microsystems, Switzerland) and a camera that had been integrated with Leica DM750 microscope. Photographs were taken randomly with a total of ten visual fields per one tissue and standardized using a global white balance. The brown color intensity was calculated using the plugin program in Image J, IHC profiler, ^24^ which will quantify the intensity of the images. The results of quantification were converted into H-Score ^25^ based on the formula:

(% low positive × 1) + (% positive × 2) + (% high positive × 3).

### Statistical analysis

Data collection was entered into the main table using Microsoft Excel 2013 (Microsoft Corporation). The tabulated data were analyzed using the Statistical Package for Social Sciences (SPSS) version 20 and visualized using GraphPad Prism 8 (RRID: SCR_002798). Data analysis was with the use of One-Way Analysis of Variance (ANOVA), followed by Duncan Post Hoc test to compare the differences. Differences of p < 0.05 are considered statistically significant. The correlation analysis between ICAM-1 expression and E-Cadherin was also analyzed using Pearson Correlation. The stronger the correlation, the closer the correlation coefficient value (r) is to 1 or −1. A positive r indicated a directly proportional correlation, while a negative r indicated an inverse correlation. The correlation model of the two biomarkers was also analyzed by simple linear regression.

## Results

**Figure 1.**
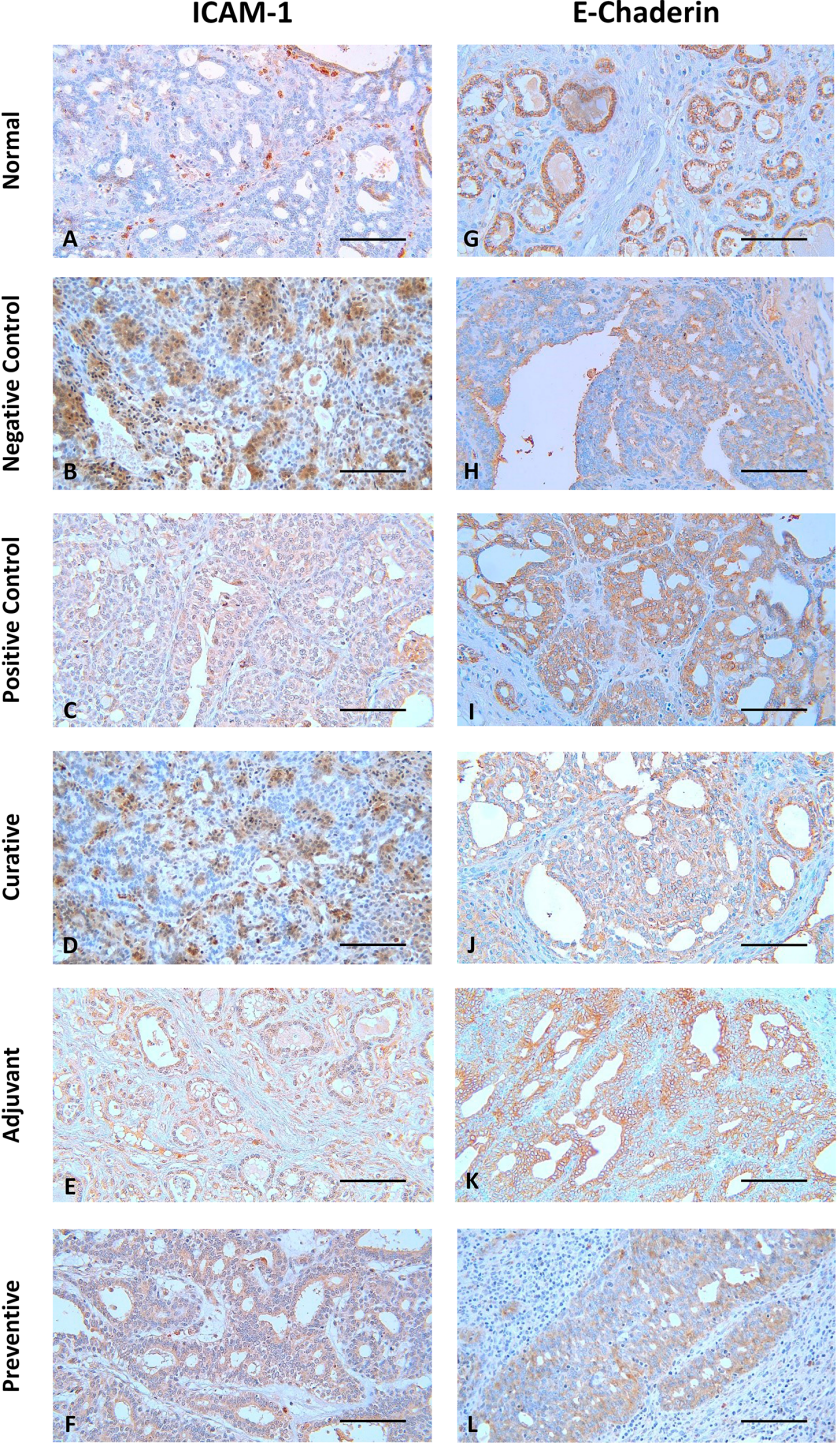
Representative image of immunohistochemistry staining for ICAM-1 and E-Cadherin in mouse breast tissue. Scale bar represents 50 μm. All images were taken at 400× magnification.

The representative image of IHC staining for ICAM-1 and E-Cadherin in breast tissue is shown in Figure 1. Each image depicts a different expression based on the group and the biomarker (ICAM-1 and E-Cadherin). Some images, such as Figure 1 B, D, F, G, I, K, and L, show a greater brown color intensity than others, which suggests that the associated biomarker is expressed at a greater level.

**Figure 2.**
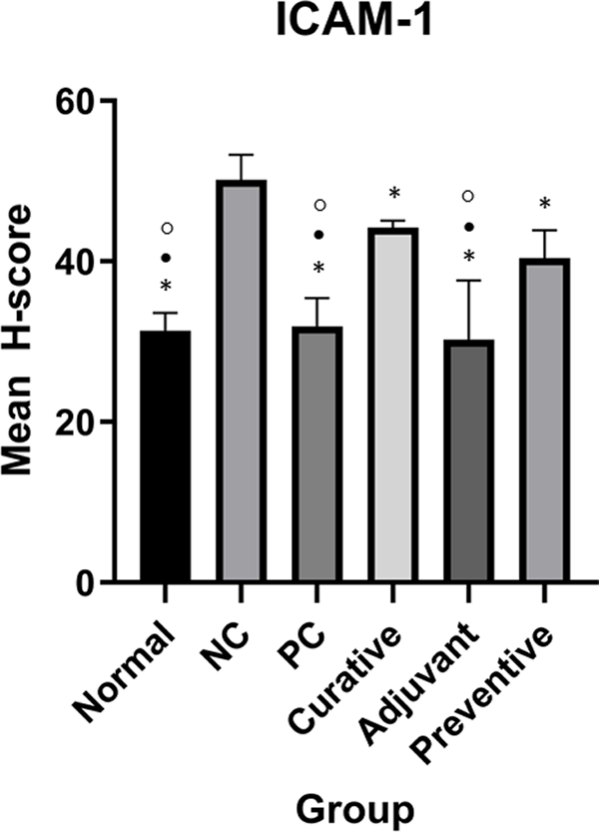
Effect of ICAM-1 in all groups. Normal, positive control (PC), curative, adjuvant, and preventive groups have significantly different ICAM-1 expressions compared to the negative control (NC). Duncan Test was used to determine the difference between groups. * represents significant difference (p < 0.05) of groups versus NC. ○ represents a significant difference (p < 0.05) of groups versus preventive.

**Figure 3.**
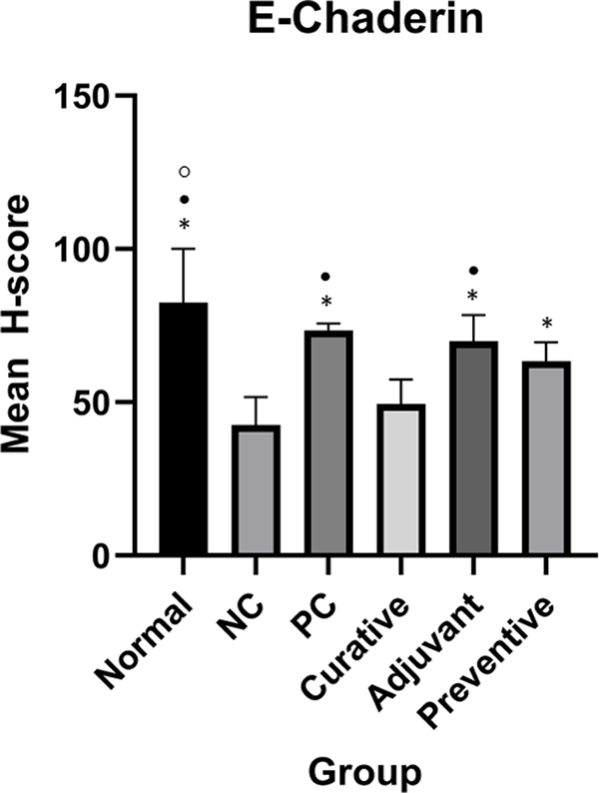
Expression of E-Cadherin in all groups. Duncan Test was used to determine the difference between groups. * represents significant difference (p < 0.05) of groups compared to NC. • represents significant difference (p < 0.05) of groups compare to curative. ○ represents a significant difference (p < 0.05) of groups compare to preventive.

However, qualitative evaluation alone is insufficient for reaching a conclusion. As a result, H-score quantification was used to quantify the quantitative expression of ICAM-1 and E-Cadherin. Table 1 shows the mean H-score in every group on ICAM-1 and E-Cadherin. Complete H-score data can be accessed in the extended data. ^26^ Figures 2 and 3 show ICAM-1 and E-Cadherin mean H-score differences between groups, respectively.

**Table 1.** ICAM-1 and E-Cadherin Mean H-score.

Group	N	ICAM-1			E-Cadherin		
		Mean H-score	SD	95% CI	Mean H-score	SD	95% CI
Normal	5	31.38	2.19	27.89-34.88	82.57	17.44	54.81-110.33
Negative control	5	50.17	3.13	45.19-55.14	42.52	9.17	27.93-57.12
Positive control	5	31.91	3.53	29.29-37.54	73.99	2.32	69.70-77.09
Curative	5	44.25	0.85	42.89-45.59	49.67	8.12	36.46-62.29
Adjuvant	5	30.25	7.38	18.51-41.99	69.96	8.42	56.57-83.36
Preventive	5	40.41	3.48	34.88-45.94	63.38	6.21	53.50-73.25

CI, Confidence Interval; N, sample size; SD, standard deviation.

One-Way ANOVA for ICAM-1 showed significant results (p < 0.001). Meanwhile, Duncan’s test results showed that there were significant differences in ICAM-1 expression between adjuvant and NC, normal and NC, PC and NC, adjuvant and preventive, normal and preventive, PC and preventive, adjuvant and curative, normal and curative, PC and curative as seen in Figure 2.

One-Way ANOVA analysis for E-Cadherin indicated significant results (p < 0.001). Meanwhile, Duncan’s test results showed there were significant differences between preventive and NC, adjuvant and NC, PC and NC, normal and NC, adjuvant and curative, PC and curative, normal and curative, normal and preventive as seen in Figure 3.

**Figure 4.**
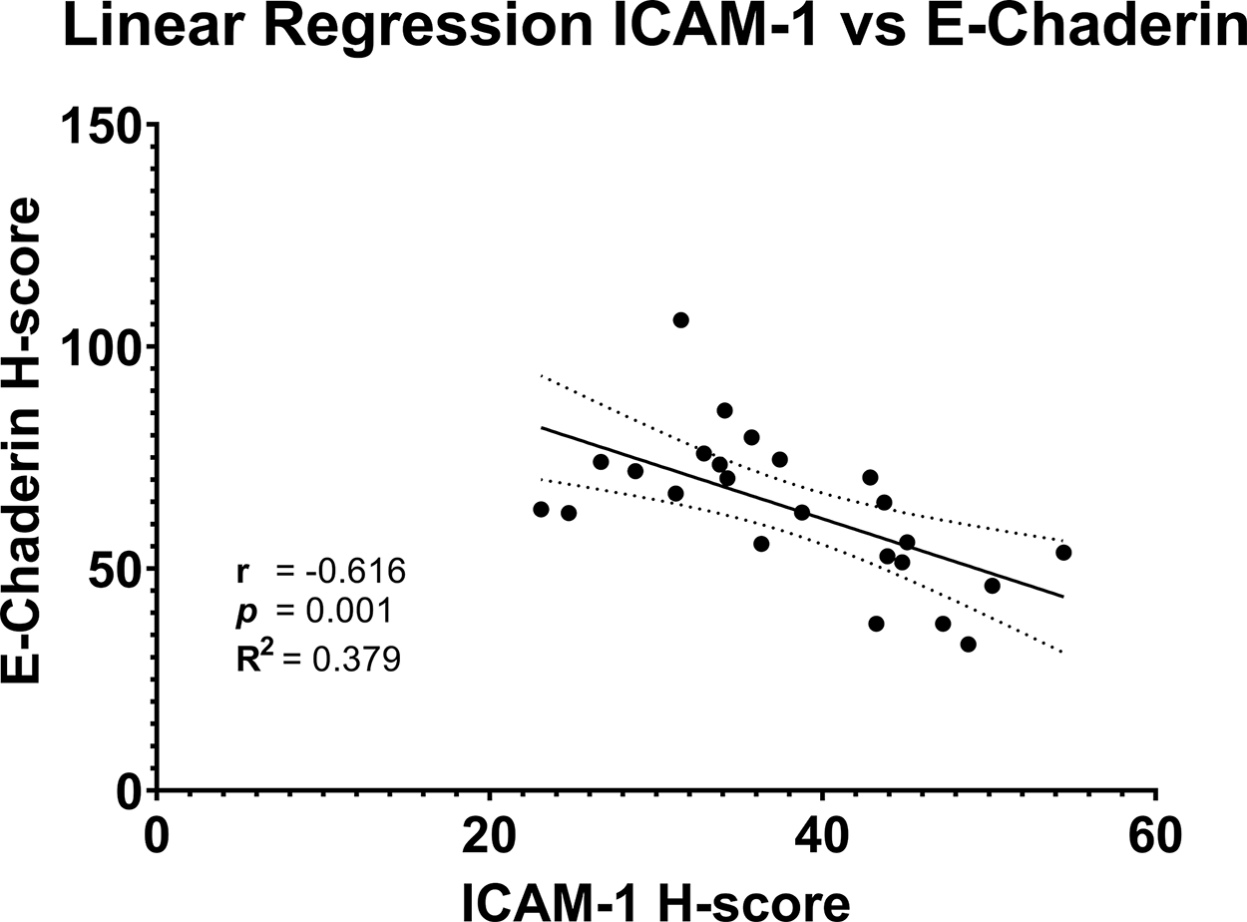
Correlation between ICAM-1 and E-Cadherin expression.

The results of the Pearson correlation analysis showed r = −0.616 (−0.8165; −0.283) with p = 0.001. The correlation between ICAM-1 and E-Cadherin expressions was negatively correlated with moderate strength. Further regression analysis produces a correlation model between E-Cadherin and ICAM-1 expressions through the equation y = −1.214x + 109.7, where y is E-Cadherin expression and x is ICAM-1 expressions, as seen in Figure 4.

## Discussion

In this study, the relationship between LE administration in the DMBA-induced breast cancer model was assessed. ICAM-1 and E-Cadherin, molecules involved in breast cancer adhesion and progression, were evaluated in each group to determine their effect. In the breast cancer model, results revealed an inverse correlation between ICAM-1 and E-Cadherin expressions. Additionally, Tamoxifen, adjuvant LE, and preventative LE all had a significant effect on either lowering ICAM-1 expression or promoting E-Cadherin expression.

DMBA as a breast carcinogen is commonly used to simulate mammary carcinogenesis for tumor research. ^27, 28^ In this study, DMBA had a substantial influence on the expression of ICAM-1 and E-Cadherin. As demonstrated in Figures 2 and 3, there is a substantial difference between NC and normal tissue samples. DMBA-induced breast models showed a significantly increased ICAM-1 expression. This is consistent with the fact that ICAM-1 expression rises with cancer progresses, ^29^ especially during metastasis, ^30^ although it is expressed at lower levels in normal tissues. ^31^ Several studies have also investigated DMBA as a tumor model and discovered a significant increase in ICAM-1 expression. ^32- 34^ Although the evidence is inconclusive, the nuclear factor-kappaB (NFkB) pathway is considered to play a role in DMBA-induced ICAM-1 upregulation. ^35, 36^ DMBA is able to potentiate NFkB ^37, 38^ via Ras mutation in the Ras/Raf/mitogen-activated protein kinase/ERK kinase (MEK)/extracellular-signal-regulated kinase (ERK) pathway, ^39, 40^ thereby increasing ICAM-1 expression. ^35, 36^ DMBA, however, decreased E-Cadherin expression. This is consistent with the hypothesis that E-Cadherin is expressed in normal cells, with its absence being a hallmark of EMT and a more migratory, invasive cancer. ^41, 42^ These results are also consistent with other research that demonstrate a reduction in E-Cadherin expression following DMBA induction. ^42, 43^ DMBA's impact on E-Cadherin, like that of ICAM-1, is mediated via NFkB activation ^37, 38^ via the Ras-Raf-MEK-ERK pathway ^39^ thus causing E-Cadherin repression due to transcription factor TWIST expression. ^44^ DMBA can also activate aryl hydrocarbon receptors (AhR), promoting transcription of SNAI2 (Slug), ^45^ an E-Cadherin repressor protein. ^46^ Furthermore, the negative correlation of ICAM-1 and E-Cadherin was consistent with the functions of each molecule. Zhao et al. ^47^ has also shown that reduced expression of E-Cadherin was associated with increased expression of ICAM-1 in mice models of breast cancer.

Tamoxifen, the most established hormone therapy for breast cancer, is an estrogen receptor antagonist that operates in its active metabolite, hydroxytamoxifen, in breast cancer. ^48^ This therapy decreased ICAM-1 expression, and increased E-Cadherin expression in DMBA-induced mouse models compared to NC, as seen in Figures 2 and 3. This finding is consistent with the results of Sun et al. ^48^ who discovered that Tamoxifen administration can reduce the Toll-like receptor 4 (TLR4)/NFkB-mediated inflammatory response, resulting in a reduction in ICAM-1 expression. This process might potentially account for the rise in E-Cadherin following Tamoxifen administration. As previously stated, NFkB mediates E-Cadherin repression, ^44^ therefore, inhibiting this pathway will increase E-Cadherin expression. Furthermore, Tamoxifen could prevent the activation of Slug and Snail, the E-Cadherin protein inhibitors, by inhibiting Transforming Growth Factor Beta (TGF-β)/Smad, thus enhancing E-Cadherin expression. ^49^

In this study, three LE groups: adjuvant, curative, and preventive, had varying effects on ICAM-1 expression. At first impression, the adjuvant group appeared to have excellent outcomes since it was substantially different from NC, preventive, and curative groups, as seen in Figure 2. This demonstrates that combining LE with Tamoxifen can dramatically lower ICAM-1 expression. However, this role may be attributed only to Tamoxifen, implying that the addition of LE had no meaningful effect on decreasing ICAM-1 expression. This was further confirmed as no significant difference between the adjuvant and PC group was found. As a result, an assessment of the effects of LE in the curative and preventive groups is essential. Figure 2 shows that the curative group did not have significant outcomes in terms of ICAM-1 expression. Zhu et al. ^50^ reported the same result, indicating that lunasin administered to monocytes was unable to decrease ICAM-1 expression. On the other hand, the preventive group showed some intriguing findings. Figure 2 shows a substantial difference in ICAM-1 expression between the preventive group with the other groups including NC, PC, and normal groups. This indicates that, even though LE at preventive doses considerably reduced ICAM-1 expression relative to NC, it was not as low as in the normal or PC groups. Although there has not been any research yet to show the preventive effect of LE on ICAM-1 expression, these findings may be explained by lunasin's antimitotic activity. A study by Hsieh et al. ^51^ found that lunasin had an anti-mitotic effect by suppressing mutations in a DMBA-induced mouse model. Jeong et al. ^52^ also shown that lunasin can prevent Ras mutations by inhibiting core H3 and H4 histone acetylation. This evidence explains why LE administered preventively (before DMBA induction) was more effective than LE administered curatively (after DMBA induction) in decreasing ICAM-1 expression. This is due to LE's ability to prevent the Ras mutation induced by DMBA, therefore preventing the NFkB pathway from being potentiated, which can enhance ICAM-1 expression.

**Figure 5.**
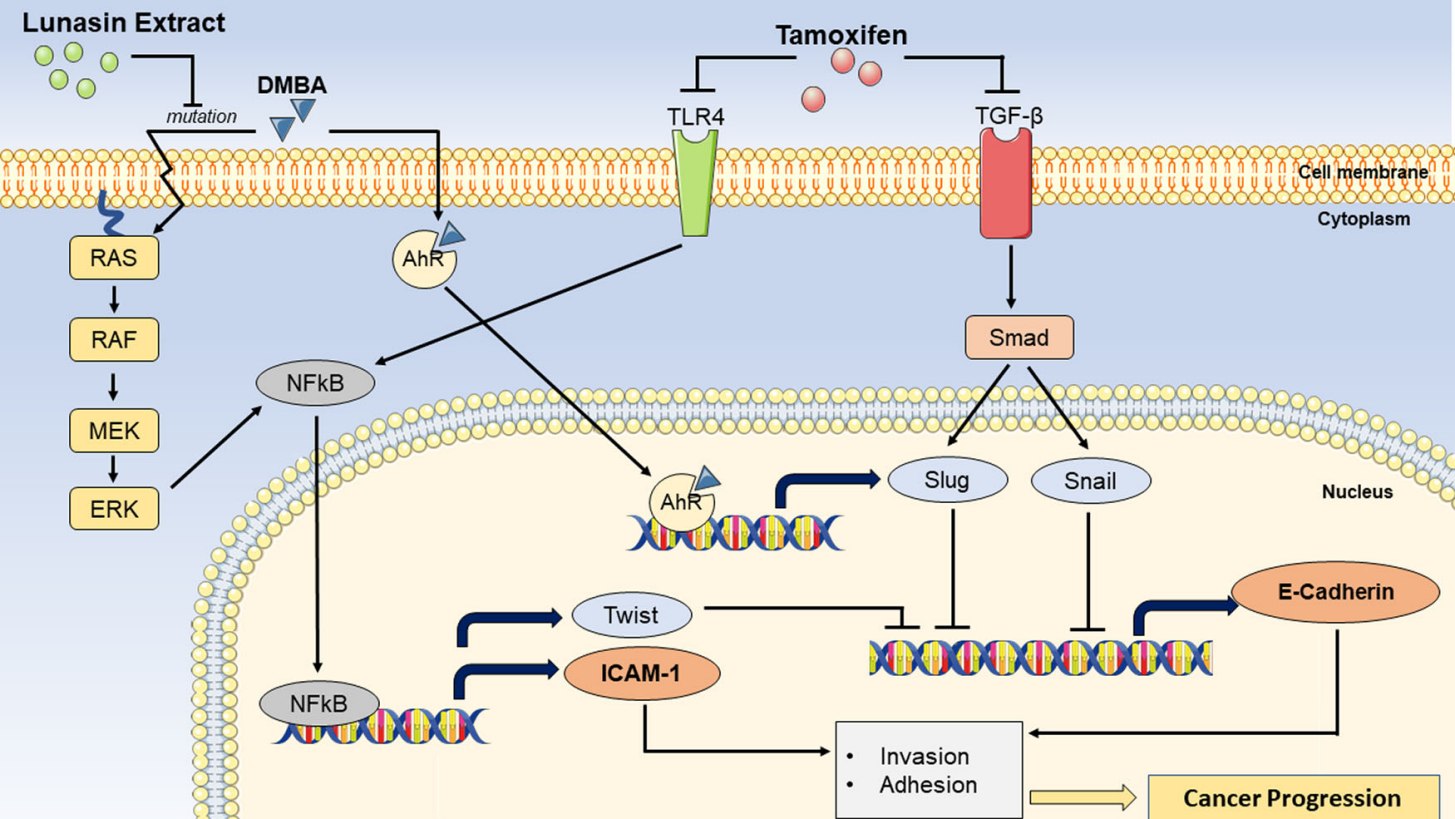
Schematic molecular pathway of LE regarding ICAM-1 dan E-Cadherin expression. 7,12-Dimethylbenz [a] anthracene (DMBA) can cause breast cancer by inducing Ras mutations, which cause nuclear factor-kappaB (NFkB) potentiation via the Ras-RAF-MEK-ERK pathway. ICAM-1 and TWIST expression will be enhanced as a result of NFkB activation. TWIST is a transcription factor that inhibits the production of E-Cadherin. DMBA can also bind to aryl hydrocarbon receptor (AhR) and promote the transcription of Slug, an inhibitor of E-Cadherin production. Lunasin extract suppresses Ras mutations, which contribute to the prevention of breast cancer progression. As a result, NFkB potentiation does not occur, resulting in a decrease in ICAM-1 expression, and an increase in E-Cadherin expression. Tamoxifen, on the other hand, can suppress Toll-like receptors (TLR)-4 and Transforming Growth Factor Beta (TGF-β). TLR-4 suppression may also prevent NFkB potentiation. Furthermore, TGF-β inhibition will disrupt the smad/Slug and smad/Snail pathways, resulting in inhibition of E-Cadherin production.

The three LE groups also exhibited distinct effects on E-Cadherin expression. The adjuvant group was able to significantly increase E-Cadherin expression as represented by the significant difference it has with both NC and curative ( Figure 3). However, similar to ICAM-1, this result might be attributed to the impact of Tamoxifen alone. Contrastingly, the curative group did not demonstrate a significant increase in E-Cadherin expression. A study by Pabona et al. ^53^ reported that the lunasin administration promoted E-Cadherin expression. However, their study is an *in vitro* study using synthetic lunasin, which is different from this study. The preventive group, unlike the curative group, showed a significant difference in E-Cadherin expression compared with the normal and NC group. However, this was not seen with the PC group. This suggests that administering a preventive dosage of LE in the breast cancer model was able to significantly enhance E-Cadherin expression relative to NC until it was as high as PC, but not as high as the normal group. Interestingly, unlike ICAM-1, E-Cadherin expression in the preventive group did not differ from that of the Tamoxifen group. This is possible because, as seen in Figure 5, more pathways influence E-Cadherin expression than ICAM-1. This creates increased heterogeneity in the expression of E-Cadherin, which is difficult to identify in this study. Aside from these differences, the preventive group outperformed the curative group in terms of enhancing E-Cadherin expression, compared to the findings on ICAM-1 expression. This finding could be explained by the same mechanism that occurs in ICAM-1, namely mutation-inhibiting action by LE. ^51, 52^

This study has limitations, such as administrating one dose (500mg/kg BW) of the LE, therefore, the effects could not be compared in a dose-dependent manner. As a result, research with a more diversified dose is recommended. Furthermore, more study on other biomarkers, both in the same and distinct molecular pathways, is required to explore the preventive potential of LE in breast cancer.

## Conclusion

A preventive dose of LE was able to reduce ICAM-1 expression while increasing E-Cadherin expression. Our findings show that LE has cancer-preventive effects in a well-characterized animal model of breast cancer. Further study, incorporating more biomarkers, is required to explore the mechanism of action of this peptide on mammary carcinogenesis as well as other forms of cancer.

## Data availability

### Extended data

(Harvard Dataverse): The potential of lunasin extract for the prevention of breast cancer progression by upregulating E-Cadherin and inhibiting ICAM-1.

DOI: https://doi.org/10.7910/DVN/EXUSQ2. ^26^

This project contains the following extended data:

• ICAM-1 Raw Data.tab: The ICAM-1 raw data is the result of the IHC profiler quantification.
• ICAM-1 Grouping.tab: grouping the results of ICAM-1 raw data based on the treatment group (normal, negative control, positive control, adjuvant, curative, and preventive).
• E-Cadherin Raw Data.tab: The result of the IHC profiler quantification.
• E-Cadherin Grouping.tab: grouping the results of E-Cadherin raw data based on the treatment group (normal, negative control, positive control, adjuvant, curative, and preventive).
• ICAM-1 and E-Cadherin Research Protocol.pdf: The experimental protocols were approved by the Ethics Committee of the Faculty of Medicine, University of Indonesia with protocol number KET-647/UN2.F1/ETIK/PPM.00.02/2019.

Data are available under the terms of the Creative Commons Zero “No rights reserved” data waiver (CC0 1.0 Public domain dedication).

## Reporting guidelines

(Harvard Dataverse): ARRIVE checklist for “The potential of lunasin extract for the prevention of breast cancer progression by upregulating E-Cadherin and inhibiting ICAM-1”.

DOI: https://doi.org/10.7910/DVN/EZTWXY. ^23^

This project contains the following extended data:

• ARRIVE 2.0 Checklist_ICAM-1 and E-Cadherin.pdf

Data are available under the terms of the Creative Commons Zero “No rights reserved” data waiver (CC0 1.0 Public domain dedication).

## Author contributions

KK, NKR, AMM designed the concept of this research article. KK, EW, NKR, AMM performed the methodology. KK carried out the overall reproducibility of experiments and other research outputs. Application of statistical, mathematical, computational, or other formal techniques to analyze or synthesize study data was done by EW. Conducting the research and investigation process, specifically performing the experiments, or data/evidence collection was done by EW, NKR, AMM. The resources were provided by KK, AE, HS. The Organization and management of the datasets collection, as well as writing the original draft of this article was by EW. Review & Editing was done by KK, NKR, AMM, AE, HS. Verification, of the overall reproducibility of experiments and other research outputs was performed by KK, AMM, AE, HS.
