# Supplementary material for: The potential of lunasin extract for the prevention of breast cancer progression by upregulating E-Cadherin and inhibiting ICAM-1
Source: F1000Res. 2021 Sep 8;10:902. [Version 1] doi: 10.12688/f1000research.55385.1 (PMC8506221; doi:10.12688/f1000research.55385.1)
Supplement: Peer review history

# Peer review: Reviewer response for version 1

**DOI:** 10.5256/f1000research.58953.r93818

**Published:** 2021-10-11

**Authors:** Hardhono Susanto

**Affiliations:** 1 Medicine Faculty, Diponegoro University, Semarang, Indonesia

This study examines the potential of Lunasin Extract (LE) as a natural product that can inhibit breast cancer progression through the biomarker mechanism. This study used biomarkers E-Chaderin and ICAM-1 as markers in evaluating the action of LE. This study used immunohistochemistry methods for assessing these biomarkers. This study indicates that LE can inhibit breast cancer progression by increasing E-Chaderin and decreasing ICAM-1.

**Some points that need to be addressed and clarified:**

1. The author needs to explain the origin of the use of LE in this study. Usually, in researching natural products, a minimum of 3 different doses will be used. If the author has conducted previous studies regarding the dose of this LE, it can be added to this study.
2. The method used in this research is excellent and complete. As input, the results of the extraction and characterization of LE may be attached. This could be very helpful for other researchers who want to create LE with different parameters.
3. The addition of Figure 5 is excellent and complete. Still, its placement in this study is more suitable as a hypothesis for further research.

**General conclusion:**

 The content of this study has linearity with the authors’ expertise. They cite enough recent literature, clearly cited. The method contains adequate and sufficient details, e.g. sum of samples, the procedure of laboratory examination with a fit statistical method. They explain their result and discussion in good enough clearly their interpretation. The statement of the conclusion answers the aim of the study. Also, in reasonable and appropriate academic language.

Is the work clearly and accurately presented and does it cite the current literature?

Yes

If applicable, is the statistical analysis and its interpretation appropriate?

Yes

Are all the source data underlying the results available to ensure full reproducibility?

Yes

Is the study design appropriate and is the work technically sound?

Yes

Are the conclusions drawn adequately supported by the results?

Yes

Are sufficient details of methods and analysis provided to allow replication by others?

Yes

Reviewer Expertise:

Biomedical and Sports Medicine

I confirm that I have read this submission and believe that I have an appropriate level of expertise to confirm that it is of an acceptable scientific standard.

# Peer review: Reviewer response for version 1

**DOI:** 10.5256/f1000research.58953.r93820

**Published:** 2021-09-23

**Authors:** Bambang Pontjo Priosoeryanto

**Affiliations:** 1 Division of Veterinary Pathology, Faculty of Veterinary Medicine, IPB University, Bogor, Indonesia

This work is an interesting study about the potential of breast cancer therapeutic substance of lunasin from a natural product of soybean. The result is very clear and indicated the potential promising of lunasin to be a candidate for combating breast cancer disorder. 

 Some points need to be addressed and clarified:

1. The work on extraction in the methods is very clear but there is no result information about the extract. Maybe it's better if there is a short piece of information about the extraction result, even if this extraction work will be published elsewhere. 
2. There is no information on how the author chose the dose of lunasin (500 mg/BW), what is the basic reason for this choice?
3. It will be better if the type of cancer was also explained in the result by at least using a general hematoxylin-eosin staining method, it will enhance the understanding of the action of lunasin in the tumor type.
4. Need clarification in the method of IHC, concerning the counterstain. it was written that used hematoxylin-eosin (it means hematoxylin and eosin) or only hematoxylin because hematoxylin will stain nuclear, while eosin stains cytoplasm.
5. Figure 5 as a concept of hypothetical lunasin action is a good direction idea for further study for the clarification of the actions cascade. 

General conclusion :

 This work is a good study in order to elaborate the lunasin activity especially as an anticancer in the breast cancer model and will have a good impact on the development of therapeutic anticancer molecules although further study is needed. Results from this study have enriched the current information about lunasin in order to better understanding the lunasin activity and its molecular mechanism.

Is the work clearly and accurately presented and does it cite the current literature?

Yes

If applicable, is the statistical analysis and its interpretation appropriate?

Yes

Are all the source data underlying the results available to ensure full reproducibility?

Yes

Is the study design appropriate and is the work technically sound?

Yes

Are the conclusions drawn adequately supported by the results?

Yes

Are sufficient details of methods and analysis provided to allow replication by others?

Partly

Reviewer Expertise:

Cancer Pathology, Anticancer activity from natural substances

I confirm that I have read this submission and believe that I have an appropriate level of expertise to confirm that it is of an acceptable scientific standard.

# Peer review: Reviewer response for version 1

**DOI:** 10.5256/f1000research.58953.r94586

**Published:** 2021-09-22

**Authors:** Asmi Chakraborty

**Affiliations:** 1 Department of Translational Medicine, Translational Glycobiology Institute at FIU, Herbert Wertheim College of Medicine, Florida International University, Miami, FL, USA

This article offers a novel therapeutic molecule, lunasin, which can work synergistically with the current treatment option of Tamoxifen, in breast cancer. While the findings are exciting and merit investigation, the following points need to be addressed:

**Major:**

1. Include more details on the breast cancer type and what DMBA induces. DMBA is not spoken about before the methods. Since this is the only major experiment there needs to be more reasoning on why this was chosen. It is known in the literature that DMBA induced rat tumors are not invasive, have longer durations of latency. Hence, further justification of the model with help the reader.
2. The authors mention that the cost of production of LE is expensive. Is the method used by the paper a cheaper way of producing it or are the authors simply mentioning this to state that this is a potential drawback?
3. Although the authors mention in the conclusion that the lack of data on dose-dependent response is a drawback, there needs to be a reason for choosing the current dosage in the study. Perhaps a reference or any other justification. This is critical.
4. The results need to be more elaborate. Did the authors perform any survival study on the animal groups?
5. There are several unestablished connections being made in the discussion. While all the pathways are valid, connecting LE to the whole carcinogenic cascade is an over-interpretation of one experiment in the paper. In my opinion, this is not appropriate and needs further experimentation to be proven. The rat tumors can be homogenized to isolate RNA and protein to further validate the claims made in the discussion. Without this validation, the authors cannot claim the statements in the discussion as it is all speculative with regards to LE.
6. Per the previous comment Figure 5. Should be a hypothesis or future direction-related figure. These are great future directions for the study.

Overall, this is a promising study that can lead to the development of synergistic treatment with current therapy for breast cancer. While further investigation is needed, this paper does offer exciting insights into the role of LE as a therapeutic molecule in breast cancer treatment.

Is the work clearly and accurately presented and does it cite the current literature?

Yes

If applicable, is the statistical analysis and its interpretation appropriate?

Yes

Are all the source data underlying the results available to ensure full reproducibility?

Yes

Is the study design appropriate and is the work technically sound?

Yes

Are the conclusions drawn adequately supported by the results?

Partly

Are sufficient details of methods and analysis provided to allow replication by others?

Partly

Reviewer Expertise:

Cancer, Glycobiology,

I confirm that I have read this submission and believe that I have an appropriate level of expertise to confirm that it is of an acceptable scientific standard, however I have significant reservations, as outlined above.
